# IQAGPT: computed tomography image quality assessment with vision-language and ChatGPT models

**DOI:** 10.1186/s42492-024-00171-w

**Published:** 2024-08-05

**Authors:** Zhihao Chen, Bin Hu, Chuang Niu, Tao Chen, Yuxin Li, Hongming Shan, Ge Wang

**Affiliations:** 1 Institute of Science and Technology for Brain-Inspired Intelligence, Fudan University, Shanghai, 200433 China; 2 Department of Radiology, Huashan Hospital, Fudan University, Shanghai, 200040 China; 3 Biomedical Imaging Center, Center for Biotechnology and Interdisciplinary Studies, Department of Biomedical Engineering, Rensselaer Polytechnic Institute, Troy, NY 12180 US; 4 MOE Frontiers Center for Brain Science, Fudan University, Shanghai, 200032 China; 5 Key Laboratory of Computational Neuroscience and Brain-Inspired Intelligence (Ministry of Education), Fudan University, Shanghai, 200433 China

**Keywords:** Deep learning, Medical imaging, Image captioning, Multimodality, Large language model, Vision-language model, GPT-4, Subjective evaluation

## Abstract

Large language models (LLMs), such as ChatGPT, have demonstrated impressive capabilities in various tasks and attracted increasing interest as a natural language interface across many domains. Recently, large vision-language models (VLMs) that learn rich vision–language correlation from image–text pairs, like BLIP-2 and GPT-4, have been intensively investigated. However, despite these developments, the application of LLMs and VLMs in image quality assessment (IQA), particularly in medical imaging, remains unexplored. This is valuable for objective performance evaluation and potential supplement or even replacement of radiologists’ opinions. To this end, this study introduces IQAGPT, an innovative computed tomography (CT) IQA system that integrates image-quality captioning VLM with ChatGPT to generate quality scores and textual reports. First, a CT-IQA dataset comprising 1,000 CT slices with diverse quality levels is professionally annotated and compiled for training and evaluation. To better leverage the capabilities of LLMs, the annotated quality scores are converted into semantically rich text descriptions using a prompt template. Second, the image-quality captioning VLM is fine-tuned on the CT-IQA dataset to generate quality descriptions. The captioning model fuses image and text features through cross-modal attention. Third, based on the quality descriptions, users verbally request ChatGPT to rate image-quality scores or produce radiological quality reports. Results demonstrate the feasibility of assessing image quality using LLMs. The proposed IQAGPT outperformed GPT-4 and CLIP-IQA, as well as multitask classification and regression models that solely rely on images.

## Introduction

In recent years, there have been many advances in the field of large language models (LLMs). LLMs such as PaLM [1], LLaMA [2], and GPTs [3–5] have shown excellent results in natural language processing, including language translation, question answering, and text generation. The most remarkable breakthrough is ChatGPT, which was built upon InstructGPT [6] using labeler-written prompts and reinforcement learning from human feedback [7]. However, LLMs such as ChatGPT are unable to process visual information as they are trained only on textual data. To address this gap, vision-language models (VLMs) [8–14], which synergistically combine the capabilities of LLMs with visual processing, were proposed to capture rich vision-language correspondence. These perform well in various multimodal tasks such as report generation, diagnosis, and vision question answering. In this context, OpenAI launched its new large VLM called GPT-4 [15], with amazing performance on multimodal tasks during dialogues. In addition, MiniGPT-4 [16] integrates an advanced LLM, Vicuna [17], and a pre-trained ViT [18] with a single linear projection layer, leading to performance close to that of GPT-4.

While LLMs and VLMs are powerful in many tasks, few efforts have been made to adapt them for image quality assessment (IQA), which is essential in the development of image reconstruction or enhancement algorithms [19–22]. In medical imaging, IQA plays a crucial role, directly influencing the accuracy and reliability of diagnoses [23, 24]. Particularly, in computed tomography (CT), reconstructed low-dose CT (LDCT) images from various deep-learning methods [25–33] may have blurring or over-smoothing problems, hindering their clinical translation. Therefore, assessing CT image quality before diagnosis is essential. Classic medical IQA methods can be either objective or subjective. Objective assessment methods use mathematical models for quantitative analysis, comparing the similarities or differences between reconstructed images and their references. Over the past decades, several objective IQA metrics have been widely used, including peak signal-to-noise ratio (PSNR), structural similarity (SSIM), and root-mean-square error (RMSE). However, these metrics are usually unsatisfactory in radiological practice, as they do not effectively reflect the diagnostic utilities of images. Subjective IQA methods, on the other hand, involve expert opinions, which more accurately reflect the clinical needs [34]. However, the continuously growing number of CT images per scan poses a major burden on radiologists, who need to carefully assess each image.

In the past few years, deep-learning methods have been developed for diverse IQA tasks, including image perception [35–38], screen content [39], video [40], and medical images [41–44]. Blind pseudo-reference image-based method [36] introduced a no-reference IQA method that creates a pseudo-reference image to facilitate the quality assessment of distorted images. Unified content-type adaptive blind IQA model [37] proposed a unified framework for assessing the quality of compressed images across different content types. However, most of these methods focus on low-level image features, ignoring high-level features, especially hierarchical semantic information that is essential in the clinical context. To address this issue, Gao et al. [44] proposed an IQA network that integrates expert knowledge, combining the overall image quality ratings of radiologists with objective metrics as training labels.

Although using overall ratings as the optimization target can well reflect the overall noise level and fidelity of the image, it cannot meet the requirements of radiologists for extraction of clinically related subtleties, such as the small blood vessels, lymph nodes, and lesions.

Recently, CLIP-IQA [45] used a CLIP model to assess the similarity between images and predefined textual prompts. However, its design for natural images and dependence on simple text prompts limit its applicability for complex medical IQA, especially in evaluating fine structures and small lesions in CT images.

**Fig. 1:**
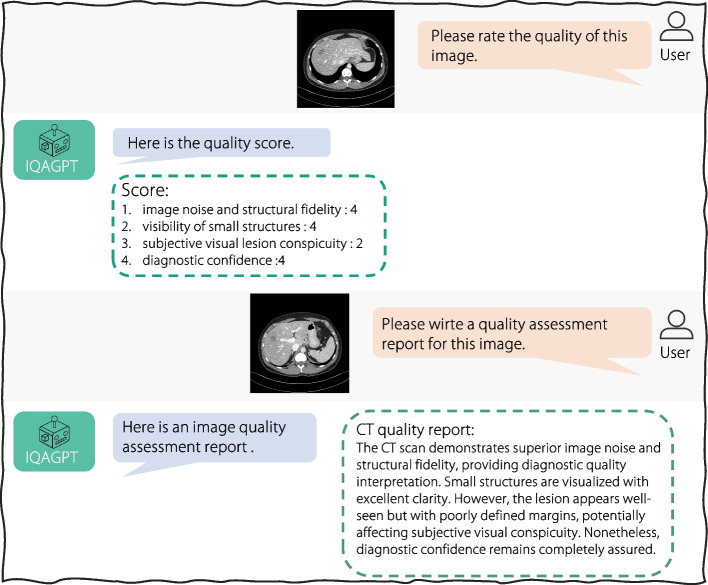
A dialogue between humans and the proposed IQAGPT. In the dialogues, IQAGPT can output scores and write the quality report based on an input image

This study developed IQAGPT, a CT IQA system based on an image-quality captioning VLM incorporated with ChatGPT to generate quality scores and summarize quality reports of CT images. First, to train IQAGPT, a dataset of 1,000 image-text pairs named CT-IQA was compiled, in which an experienced radiologist scored CT images of different qualities, similar to the subjective evaluation previously reported [28, 34]. The qualities included image noise, small structures, lesion conspicuity, and diagnostic confidence. To utilize the strengths of LLMs in subjective image evaluation, a prompt template was designed to convert the quality scores to text descriptions. Second, an image-quality captioning model built upon a pre-trained medical VLM [12] was developed and fine-tuned on the CT-IQA dataset using an autoregressive language modeling objective that predicts the next token given previous tokens [3]. Finally, through interacting with ChatGPT, IQAGPT can score CT images and generate quality reports based on the caption from the image-quality captioning model. Figure 1 presents an exemplary dialogue between a user and the proposed IQAGPT.

In summary, the main contributions of this work are as follows.

- A hybrid large model approach for CT-IQA, which synergizes the objective and subjective image quality evaluation in a clinically important scenario, is introduced.
- An IQA system consisting of VLMs and ChatGPT, termed IQAGPT, which is built on an image-quality captioning model and can output quality scores and reports by interacting with ChatGPT, is developed.
- A CT-IQA dataset for IQA, containing 1,000 image-text pairs professionally annotated according to four common subjective metrics used in diagnosis, was compiled.
- Preliminary results demonstrate the feasibility of assessing CT image quality using IQAGPT, and the resulting text-guided image-quality captioning model outperforms GPT-4 and CLIP-IQA. Furthermore, external evaluations by additional radiologists and performance on new data demonstrate the robustness and generalizability of the proposed method, respectively.

## Methods

This study aims to develop a CT IQA system, called IQAGPT, using VLMs and ChatGPT. In CT-IQA dataset subsection, the CT-IQA dataset is detailed. Thereafter, in Image-quality captioning model and Interaction with ChatGPT subsections, the image-quality captioning model and IQAGPT which interacts with ChatGPT, are described, respectively. The implementation details are presented in Implementation details subsection and the performance evaluation of IQAGPT is explained in Evaluation metrics subsection.

### CT-IQA dataset

To adapt to the IQA tasks and accurately assess the quality of CT images, an image-text dataset called CT-IQA, was compiled, in which an experienced radiologist subjectively assessed the CT images.

#### Characteristics

**Fig. 2:**
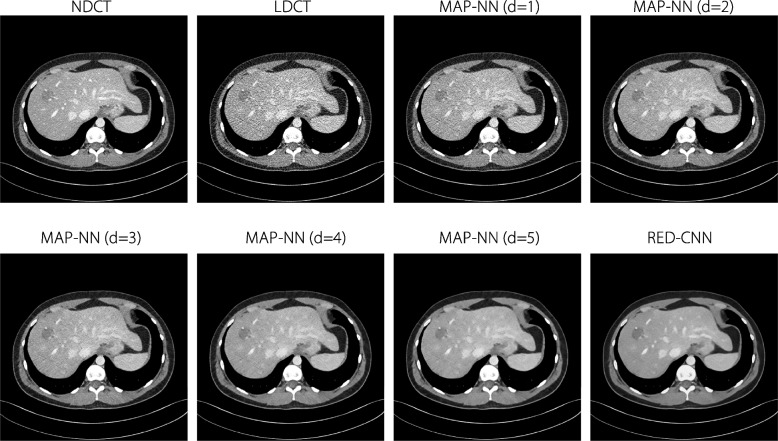
Examples of images from the CT-IQA dataset

Normal-dose CT (NDCT) slices and corresponding LDCT images at 25% of the normal dose were randomly selected from the 2016 AAPM Grand Challenge dataset [46], which includes abdominal CT scans of 10 anonymous patients. Specifically, 100 NDCT and LDCT pairs were uniformly selected from 8 patients for training and 25 slice pairs were uniformly selected from the remaining 2 patients for testing. Each scan was acquired using a Siemens SOMATOM Flash scanner and reconstructed with a B30 kernel and 1 mm slice thickness. The NDCT scans were acquired at 120 kV and 200 quality reference mAs (QRM), and the LDCT scans were acquired at 120 kV and 50 QRM. Additionally, some lesions in NDCT and corresponding LDCT were randomly simulated to evaluate subjective visual lesion conspicuity. The selected 125 LDCT images were processed using a modularized denoising model [28] called MAP-NN, producing various intermediate denoised images with associated noise reduction directions. RED-CNN [25], a widely used denoising model, was implemented, and optimized using the MSE loss function. Finally, 1,000 CT slices with different quality were obtained, including 125 NDCT slices with corresponding 125 LDCT slices, 625 reconstructed images with 5 denoising levels from MAP-NN, and 125 reconstructed images from RED-CNN. An abdomen window of all CT scans [-160, 240] HU was employed to visualize abdominal organs. These were normalized into a range of [0, 1]. Figure 2 presents CT images of eight different quality levels from the dataset, including LDCT, NDCT, images denoised with MAP-NN, and images denoised with RED-CNN.

#### Annotation process

First, a web page was created where all data were randomly displayed, including CT images of eight different levels in the dataset, including LDCT, NDCT, denoised image of MAP-NN, and denoised image of RED-CNN. Subsequently, a radiologist scored these CT images in terms of four metrics used in previous studies [28, 34], defined as follows.

- Image noise and structural fidelity on a four-point scale: 1 = better than usual, acceptable for diagnostic interpretation; 2 = average, acceptable for diagnostic interpretation; 3 = sub-optimal, for limited diagnostic information only; and 4 = unacceptable for diagnostic interpretation.
- The visibility of small structures (small blood vessels, adrenal glands, small lymph nodes) on a four-point scale: 1 = excellent visualization; 2 = acceptable visibility; 3 = sub-optimal visibility; and 4 = unacceptable visualization.
- Subjective visual lesion conspicuity (N/A = if no lesion) on a four-point scale: 1 = well-seen lesion with well-visualized margins; 2 = well-seen lesion with poorly visualized margins; 3 = poorly seen lesion with poorly visualized margins; and 4 = lesion blurred with severe loss of margins.
- Diagnostic confidence on a four-point scale: 1 = completely confident; 2 = probably confident; 3 = confident only for a limited clinical entity such as a kidney stone, a calcified lesion, or a large lesion; and 4 = poor confidence.

**Fig. 3:**
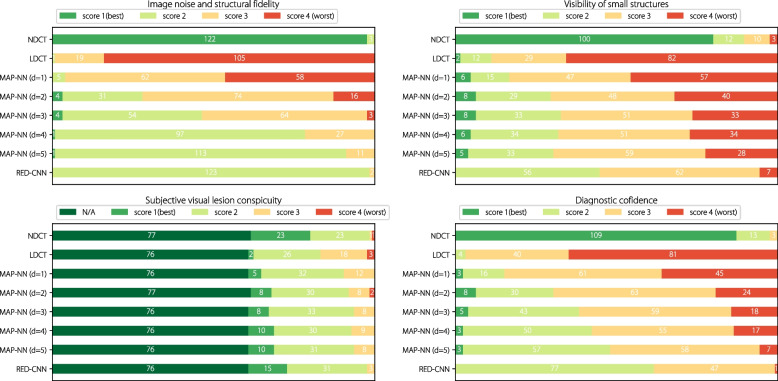
Score distribution of four metrics assessed by the radiologist in constructing CT-IQA dataset. Scores 1, 2, 3, and 4 are defined in CT-IQA dataset subsection

The scoring process was double-blinded; that is, the radiologist did not know the type of images under evaluation; NDCT was not availed for reference. Figure 3 shows the distribution of human expert scores across the aforementioned four metrics for CT images of eight different image qualities: NDCT, LDCT, MAP-NN (d = 1), MAP-NN (d = 2), MAP-NN (d = 3), MAP-NN (d = 4), MAP-NN (d = 5), and RED-CNN. The denoising level is represented by d.

The objective metrics for images of varying quality were calculated with NDCT as the reference image, shown in Table 1. RED-CNN achieves the best performance in PSNR and RMSE. However, as illustrated by the distribution of annotated scores in Fig. 3, the objective results generated by RED-CNN do not align with the professional preference, which further highlights the necessity to align the annotation of subjective quality-assessment datasets with professional preference.

**Table 1:** Quantitative performance measures on different quality levels, with NDCT as the reference image

Method	PSNR↑	RMSE↓	SSIM↑
LDCT	21.85 ± 1.25	0.082 ± 0.011	0.7897 ± 0.0250
MAP-NN (d = 1)	24.01 ± 1.26	0.063 ± 0.009	0.8171 ± 0.0229
MAP-NN (d = 2)	25.56 ± 1.25	0.053 ± 0.007	0.8339 ± 0.0215
MAP-NN (d = 3)	26.43 ± 1.22	0.048 ± 0.006	**0.8394 ± 0.0209**
MAP-NN (d = 4)	26.80 ± 1.19	0.046 ± 0.006	0.8365 ± 0.0211
MAP-NN (d = 5)	26.89 ± 1.16	0.046 ± 0.006	0.8294 ± 0.0218
RED-CNN	**27.16 ± 1.12**	**0.044 ± 0.005**	0.8270 ± 0.0225

### Image-quality captioning model

**Fig. 4:**
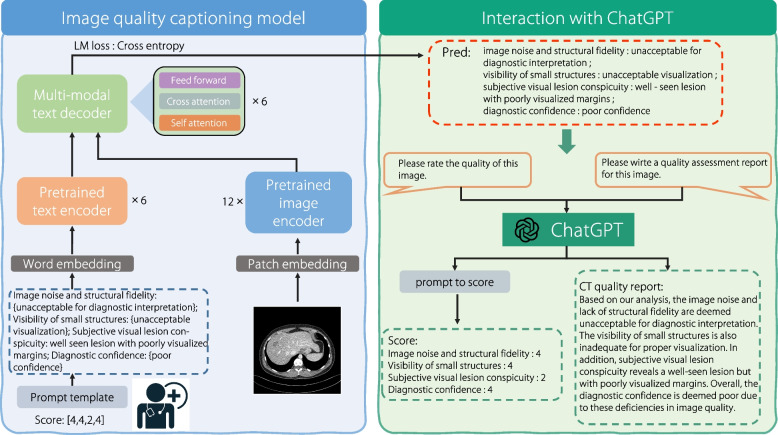
Overview of IQAGPT. While the left side shows the proposed image-quality captioning model, the right side details the process of the score and report generation through interacting with ChatGPT

Instead of using the rating scores to train the classification or regression model, an image-quality captioning model is developed to summarize the image quality. By doing so, the VLM with semantic text information and image-text fusion can better appreciate the subjective scores than image-only models—this is further discussed in Results section. The proposed model is based on a pre-trained medical VLM and fined-tuned with an autoregressive language modeling objective on the CT-IQA dataset. To leverage the capabilities of LLMs in the subjective image evaluation, we convert scores to quality descriptions using a specific prompt template during training. The prompt template is defined as “Image noise and structural fidelity: {description 1}; Visibility of small structures: {description 2}; Subjective visual lesion conspicuity: {description 3}; Diagnostic confidence: {description 4}.” Every description is the evaluation criterion corresponding to the score described in CT-IQA dataset subsection. An example of score conversion to quality caption is given in the lower left part of Fig. 4, where the score assessed by the radiologist is [4, 4, 2, 4].

The left side of Fig. 4 presents the overall framework of the proposed image quality captioning model, consisting of an image encoder, text encoder, and multimodal text decoder. The image encoder is a 12-layer visual transformer ViT-S/16 [18] while the text encoder is the first 6 layers of the BERT_base_ [47] model. The multimodal text decoder consists of the last 6 layers of BERT_base_; its role is to fuse image and text features through cross-modal attention. Some recent studies have incorporated cross-modal attention [48–50]. Min et al. [49, 50] used the normalization and summation fusion function to integrate audio-visual contexts. In contrast, the proposed captioning model leverages the synergy between visual data and textual descriptions through a transformer-based [18] cross-attention mechanism to fuse image features with text features. The image encoder, text encoder, and multimodal decoder have been pre-trained in radiography images and report pairs [12] using four widely used learning objectives in the field of vision-language alignment; more details on these four objectives are in ref. [12]. We then fine-tuned the pre-trained models to predict the next word for IQA on the CT-IQA dataset, using an auto-regressive paradigm. We hypothesize that this paradigm, combined with the proposed input template, allows LLMs to better comprehend the relationship between different metrics. CT-text pair is denoted as $(I, T)$, where $I$ represents a CT slice and $T$ is defined as $T = ({t}_{1}, {t}_{2}, . . . , {t}_{m})$ with $m$ tokens. The objective function is defined as follows:

1 $$\begin{array}{c}L\left(I,T\right)=-\left(\text{log}P\left({t}_{1}|I;\theta \right)+\sum_{i=2}^{m}\text{log}P\left({t}_{i}|{t}_{1:i-1},I;\theta \right)\right)\end{array}$$

where ${t}_{i}$ is the next token to be predicted and ${t}_{1:i-1} = ({t}_{1}, {t}_{2}, . . . , {t}_{i-1})$ represents the sequence of all previous tokens. $P$ is the conditional probability modeled by the image-quality captioning model, and $\theta$ represents the trainable parameters of the model.

### Interaction with ChatGPT

ChatGPT provides a language interface with remarkable reasoning capabilities across many domains [11]. The proposed IQAGPT enables the interaction between ChatGPT and users to generate more comprehensive output information, as depicted on the right side of Fig. 4. When users upload CT images, they can prompt IQAGPT with requests like “Please rate the quality of this image.” or “Please write a quality-assessment report for this image.” Subsequently, the users receive either quality scores or detailed quality reports. To this end, ChatGPT is used to perform corresponding operations on the output caption from the image-quality captioning model. For score-related demands, it converts the predicted caption to a score according to the prompt template described in CT-IQA dataset subsection. For report-related demands, it summarizes the predicted caption into a quality-assessment report in a radiology report format.

While it is straightforward to obtain scores using a look-up table, integrating ChatGPT into the proposed model leverages its advanced natural language understanding capabilities to generate detailed and context-aware quality reports, providing the following benefits. (1) Contextual understanding: the ability of ChatGPT to comprehend and generate contextually relevant text ensures that the quality reports are not only accurate but also rich in clinical context, which is more friendly for radiologists. (2) Flexibility: unlike a static look-up table, ChatGPT can be adapted to variations in input data, providing more nuanced and flexible assessments. (3) Scalability: ChatGPT can easily incorporate new quality metrics without requiring significant modifications to the model structure.

### Implementation details

All parameters of the proposed model were fine-tuned using a 32 GB NVIDIA V100 GPU. During training, the image-quality captioning model was fine-tuned in IQAGPT for 50 epochs with a batch size of 8, in which we used the AdamW optimizer [51] and a weight decay of 0.02. The initial learning rate was 2.0 × 10^–4^, and warm-up [52] in the first 2 epochs had a learning rate of 1.0 × 10^–5^, gradually reduced to 1.0 × 10^–6^ with cosine annealing [53]. For data processing, full-size images were employed within an abdomen window of [-160, 240] HU. The data of 10 patients were split into training and testing datasets at a ratio of 8:2 as described in CT-IQA dataset subsection. The training data were augmented through horizontal flipping and rotation.

### Evaluation metrics

To show the effectiveness of IQAGPT, we quantitatively evaluated the performance of generated quality captioning and score. First, captioning results were analyzed using widely recognized metrics in text generation tasks: bilingual evaluation understudy (BLEU-n; “n” means n words) [54], recall-oriented understudy of gisting evaluation (ROUGE-L; “L” means the longest common subsequence) [55], metric for evaluation of translation with explicit ordering (METEOR) [56], and consensus-based image description evaluation (CIDEr; “r” stands for recall) [57]. These metrics measure the similarity between the generated and reference texts, with higher scores for better quality. BLEU measures the quality of machine-translated text compared to a human reference translation. It calculates the precision for the candidate sentence based on n-grams (phrases of n words) with respect to the reference texts. ROUGE-L focuses on the longest common subsequence between the evaluated text and the reference text. METEOR is based on the harmonic mean of unigram precision and recall, with recall weighted higher than precision. METEOR calculates the weighted harmonic mean of unigram precision and recall, prioritizing recall over precision. CIDEr quantifies the resemblance of the crafted sentence to multiple reference sentences, considering the agreement among human evaluators. Notably, BLEU-n, ROUGE-L, and METEOR scores range from 0 to 1 while CIDEr scores range from 0 to infinity. In addition, the output text descriptions were converted into scores, the performance was compared in terms of accuracy as the classification evaluation, and the Pearson linear correlation coefficient (PLCC) and Spearman’s rank order correlation coefficient (SROCC) were computed to evaluate the regression.

## Results

### Evaluation of generated quality captioning

**Fig. 5:**
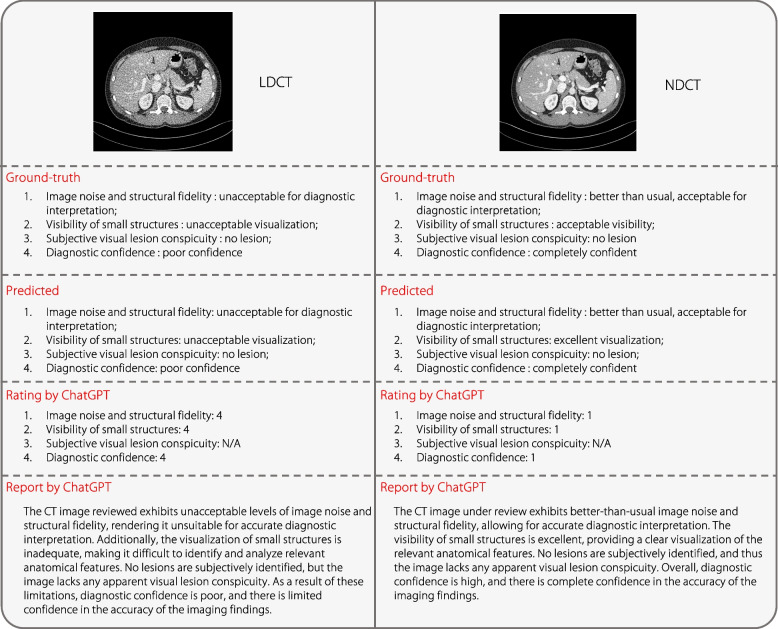
Captions predicted using the predicted method and scores and reports generated using ChatGPT

Two examples of the test results are presented in Fig. 5, where the predicted descriptions are converted to scores and quality reports using ChatGPT. It can be observed that IQAGPT consistently generates quality descriptions in excellent alignment with the annotations of radiologists. Furthermore, the reports generated using ChatGPT are consistent with the outputs from the proposed quality captioning model, which effectively overcomes the limitations of the existing VLM dialogue when assessing the quality of medical images. The quantitative captioning performance of IQAGPT and MiniGPT-4 were compared, as depicted in Table 2. GPT-4 [15] was not employed as its latest version, GPT-4 V, was not tailored for interpreting specialized medical imagery such as CT scans. The learnable linear layer in MiniGPT-4 was fine-tuned using the CT-IQA dataset in the experimental settings described by Zhu et al. [16]. IQAGPT achieves better quantitative results in seven metrics. The requirement for vast amounts of training data is a significant challenge for LLMs like MiniGPT-4. Considering the time and resource limitations, only 1,000 image-text pairs were annotated in the CT-IQA dataset to demonstrate the feasibility of the proposed method. MiniGPT-4, with its considerable size of seven billion parameters, struggles with this limited dataset, leading to unstable output and compromised performance. Conversely, the proposed IQAGPT, with a more compact model structure, produces more stable and accurate results.

**Table 2:** Quantitative evaluation of captioning quality using IQAGPT and MiniGPT-4

Method	Parameter	BLEU-1	BLUE-2	BLEU-3	BLEU-4	METEOR	ROUGE-L	CIDEr
MiniGPT-4	7B	0.798	0.733	0.717	0.652	0.516	0.826	3.070
IQAGPT	210 M	**0.819**	**0.777**	**0.742**	**0.712**	**0.546**	**0.858**	**3.620**

### Evaluation of generated quality score

**Fig. 6:**
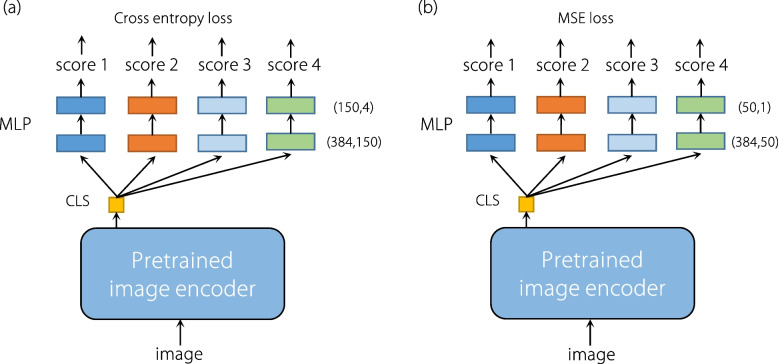
Flowcharts of (a) multi-task classification model ViT-C and (b) multi-task regression model ViT-R, respectively. CLS tokens are followed by four groups of classifiers, each consisting of two fully connected layers. Scores 1, 2, 3, and 4 are the categories corresponding to the four metrics described in CT-IQA dataset subsection

To validate the efficacy of the proposed image-quality captioning model, the proposed prompt template was employed to transform output text descriptions into scores to assess IQAGPT performance in both classification and regression tasks. A comparative study on IQAGPT was conducted with an image-only multi-task classification model, using accuracy as a metric. Additionally, IQAGPT was compared with CLIP-IQA [45] and an image-only multi-task regression model, employing PLCC and SROCC. These quantitative results are detailed in Tables 3 and 4. The PLCC and SROCC calculation for the metric of subjective visual lesion conspicuity was not performed as over half of the CT scans in the dataset did not contain lesions. The image-only multi-task classification and regression models were named ViT-C and ViT-R respectively. First, the same pre-trained image encoder (ViT-S/16) was employed in IQAGPT to extract image features. Then, four pairs of fully connected layers were implemented following the classification (CLS) token for four metrics, as depicted in Fig. 6. ViT-C and ViT-R employed cross-entropy loss and mean squared error loss respectively. The same training strategy was used with IQAGPT to train CLIP-IQA + , ViT-C, and ViT-R.

**Table 3:** Comparison of classification evaluation accuracy between IQAGPT and ViT-C

Classification	ViT-C	IQAGPT
Image noise and structural fidelity	0.545	**0.765**
Visibility of small structures	0.405	**0.620**
Subjective visual lesion conspicuity	0.725	**0.820**
Diagnostic confidence	0.375	**0.605**
Mean	0.512	**0.702**

**Table 4:** Comparison of IQAGPT with CLIP-IQA and ViT-R in the regression evaluation performance in terms of PLCC/SROCC

Regression	CLIP-IQA	CLIP-IQA +	ViT-R	IQAGPT
Image noise and structural fidelity	0.277/0.271	0.742/0.633	0.580/0.460	**0.821/0.820**
Visibility of small structures	0.121/0.117	0.712/0.696	0.436/0.415	**0.743/0.735**
Subjective visual lesion conspicuity	**-**	-	-	**-**
Diagnostic confidence	0.081/0.069	0.650/0.642	0.504/0.422	**0.699/0.689**
Mean	0.160/0.114	0.701/0.657	0.531/0.519	**0.754/0.748**

CLIP-IQA + represents the fine-tuned version of CLIP-IQA

Table 3 shows that IQAGPT outperforms the image-only classification model, ViT-C, across four metrics, achieving a notable improvement of 0.19 in mean accuracy. For regression, IQAGPT surpasses ViT-R and CLIP-IQA, as shown in Table 4. Compared with ViT-C and ViT-R, which represent ablation methods without LLM, IQAGPT outperforms them because it uses LLM to analyze detailed text information instead of using only raw scores as labels. Regarding CLIP-IQA, which uses CLIP to perceive subjective attributes through text prompt pairing, these texts contain only a single adjective, enabling the assessment of global quality attributes such as noisiness and brightness, but insufficient to capture complex details in medical images for diagnosis. In contrast, IQAGPT has complex text descriptions using an autoregressive LLM model. Furthermore, a notable advantage of IQAGPT is its efficiency; unlike CLIP-IQA, which requires separate fine-tuning for each of the four metrics, IQAGPT can simultaneously produce results for all metrics in a single output.

For each image-quality level and metric, accuracy was computed using converted scores, as depicted in Table 5. The relative accuracies associated with intermediate images generated by MAP-NN may not be highly robust owing to their similar features. This aligns with the challenges in the subjective evaluation of images with subtle quality differences, a critical aspect of the CT-IQA dataset. This study highlights the complexity of differentiating between similar images.

**Table 5:** Accuracy for each of the four metrics in eight image-quality levels

Metric	NDCT	LDCT	MAP-NN (1)	MAP-NN (2)	MAP-NN (3)	MAP-NN (4)	MAP-NN (5)	RED-CNN	Mean
Metric 1	1.000	0.800	0.600	0.520	0.480	0.880	0.880	1.000	0.765
Metric 2	0.960	0.680	0.480	0.360	0.520	0.600	0.640	0.720	0.620
Metric 3	0.920	0.920	0.880	0.760	0.800	0.840	0.760	0.800	0.820
Metric 4	0.920	0.760	0.360	0.400	0.320	0.440	0.680	0.960	0.605
Mean	0.950	0.790	0.580	0.510	0.520	0.690	0.740	0.840	0.702

Metric 1: Image noise and structural fidelity; Metric 2: Visibility of small structures; Metric 3: Subjective visual lesion conspicuity; and Metric 4: Diagnostic confidence. MAP-NN (·) provides 5 denoising levels [28]

**Fig. 7:**
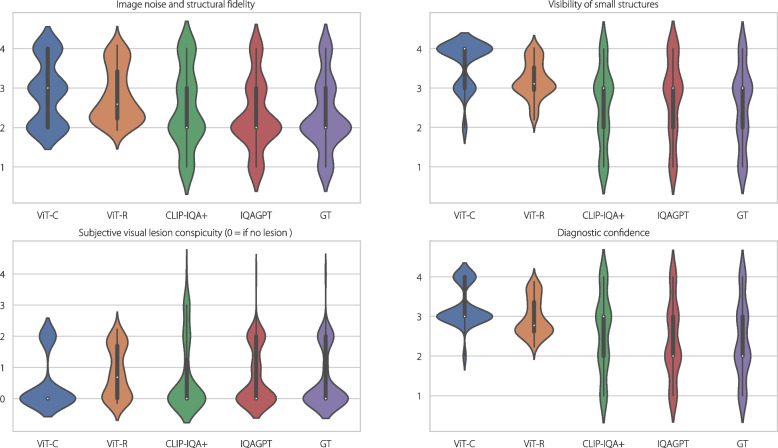
Scores distribution for four quality metrics using IQAGPT, ViT-C, ViT-R, and CLIP-IQA + . The last column lists the GT scores

Furthermore, the score distributions of IQAGPT, ViT-C, ViT-R, and CLIP-IQA + for four quality metrics are presented in Fig. 7. Notably, our method more closely approximates the groundtruth (GT) compared to ViT-C, ViT-R, and CLIP-IQA + , demonstrating its effectiveness. Overall, IQAGPT has a higher correlation with human perception than the competing methods, marking a significant advancement in CT subjective IQA.

**Fig. 8:**
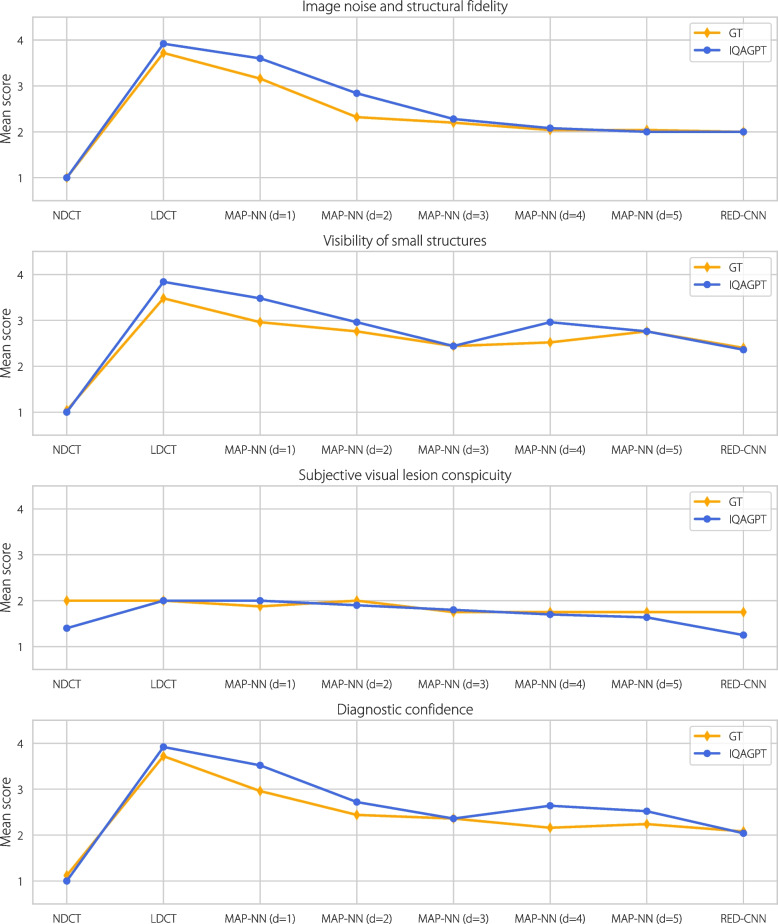
Mean values of the GT scores and the scores predicted by IQAGPT

**Fig. 9:**
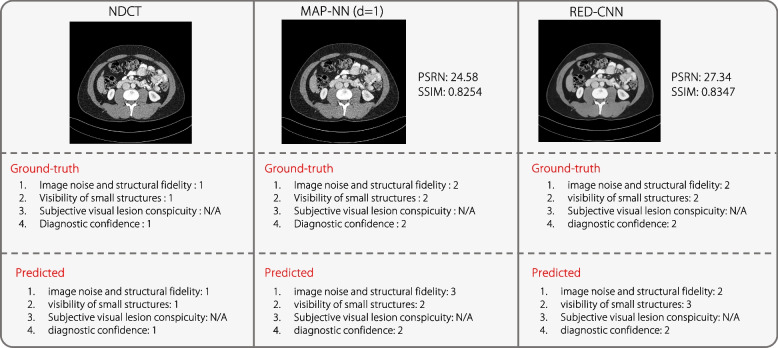
Scores of three examples predicted using IQAGPT

To further demonstrate the effectiveness of IQAGPT, the predicted and GT scores for each quality level and metric are visualized in Fig. 8, demonstrating the prediction accuracy of IQAGPT. In addition, Fig. 9 presents the predicted scores for NDCT and corresponding denoising results from MAP-NN (d = 1) and RED-CNN, along with the calculated PSNR and SSIM. It can be observed that the quantitative results of MAP-NN (d = 1) are inferior to that of RED-CNN; however, in professional subjective assessment, these two are similar and considered acceptable. From the perspective of the radiologist, the results of MAP-NN (d = 1) suffer from incomplete denoising, leading to some blurred details, while the RED-CNN results exhibit over-smoothing issues due to the use of a pixel-level loss function. In contrast, the scores of the results predicted by IQAGPT are almost identical to the GT ones, demonstrating that IQAGPT learned IQA expertise consistent with the clinical needs.

### Evaluation on new datasets

This study proposes a novel paradigm for IQA by leveraging LLMs to generate text descriptions; however, no datasets specifically annotated for this purpose currently exist. To further verify the generalizability of IQAGPT, we evaluated it on “Low Dose CT Image and Projection Data” latest released by Mayo Clinic in 2020 [58], named Mayo2020 dataset, which includes NDCT and LDCT images. Owing to the cost of professional annotations, the radiologist annotated several images.

**Fig. 10:**
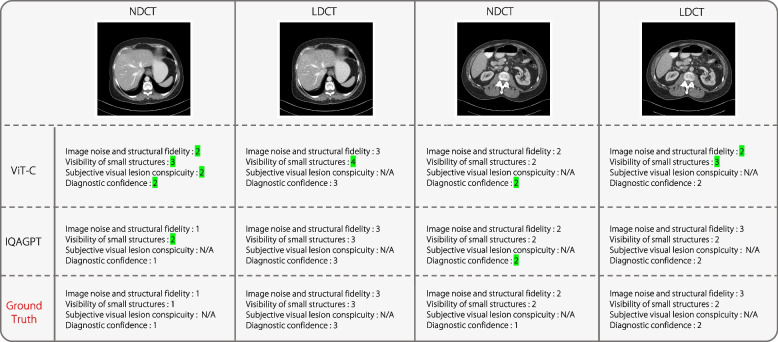
Scores of four examples predicted using IQAGPT in Mayo2020 dataset. Wrong scoring is highlighted in green

Figure 10 shows that IQAGPT is more consistent with the gold standard of the radiologist than ViT-C. Furthermore, IQAGPT did not blindly categorize NDCT as the best or LDCT as the worst. This is because the noise characteristics of the Mayo2020 differ from those of the CT-IQA dataset used for training in this study. IQAGPT produced results that align with the preference of the radiologist, demonstrating its adaptability across different datasets.

### External evaluation by additional radiologists

The CT-IQA dataset was annotated by one radiologist. To investigate the bias introduced by the radiologist, an external evaluation was conducted, in which two additional radiologists were invited to annotate. Each radiologist independently assessed the CT images using the quality metrics predefined in this study. Considering the high cost and significant time required for professional annotations, this was limited to the test dataset only. Radiologist 1 (R1), with nine years of experience, was the original annotator for both the training and testing sets in previous evaluations. Each of the two additional radiologists, Radiologist 2 (R2) and Radiologist 3 (R3), had two years of experience.

The performance of IQAGPT was evaluated separately for each radiologist’s annotations using PLCC and SROCC. Table 6 shows that the best results are achieved on the test set annotated by R1, indicating the consistency between the training and test sets annotated by R1. The PLCC/SROCC scores for R2 and R3, though dropping a little bit, are still comparable to those of R1, demonstrating the strong robustness of the developed model against external evaluation and verification. Different radiologists have different biases regarding image quality; however, the high correlation among radiologists shows a small bias between internal and external evaluations.

**Table 6:** Performance of IQAGPT with three radiologist annotations in the regression evaluation in terms of PLCC/SROCC

Regression	R1	R2	R3
Image noise and structural fidelity	**0.821/0.820**	0.750/0.756	0.755/0.750
Visibility of small structures	**0.743/0.735**	0.668/0.659	0.680/0.673
Subjective visual lesion conspicuity	**-**	-	-
Diagnostic confidence	**0.699/0.689**	0.661/0.672	0.679/0.688
Mean	**0.754/0.748**	0.694/0.696	0.705/0.704

### Ablation on LLMs

**Fig. 11:**
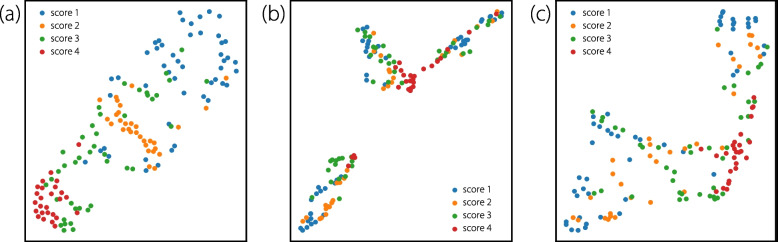
Feature visualization of the CLS token in the image encoder of (a) IQAGPT, (b) ViT-C, and (c) ViT-R, using the *t*-SNE method. The samples are labeled with categories from the metric of image noise and structural fidelity

**Fig. 12:**
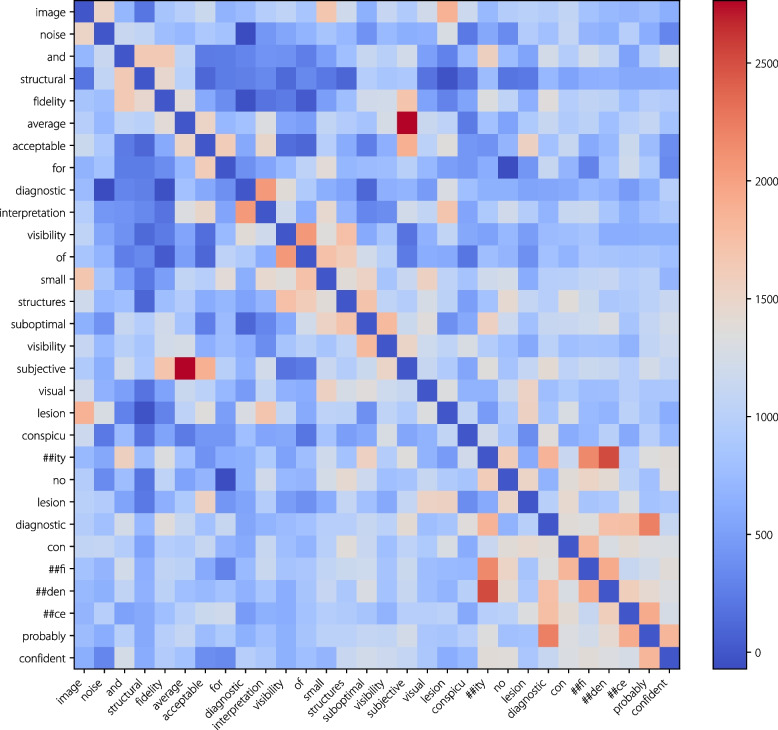
Self-attention map of tokens from the last layer in the multimodal text decoder

To further demonstrate the effectiveness of textual semantic information, the $t$-SNE [59] method was employed to visualize the features of the CLS tokens in the image encoders of IQAGPT, ViT-C, and ViT-R, as illustrated in Fig. 11. Each sample was labeled using the score of the image noise and structural fidelity metric. This visualization demonstrates that IQAGPT distinguishes features of different categories more clearly than ViT-C and ViT-R, and exhibits an ordered sequence in the score-based feature representation. Additionally, the self-attention map of tokens from the multimodal text decoder, depicted in Fig. 12, reveals that each token is interconnected not only with tokens from the same task but also with those from preceding tasks. This finding underscores the merits of textual descriptions in capturing inter-task correlations, enhancing the classification performance.

### Interpretation

**Fig. 13:**
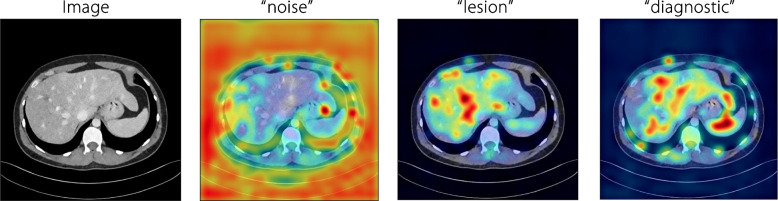
Grad-CAM visualizations on the cross-attention maps corresponding to individual words

To provide an interpretation of the proposed quality captioning model, per-word Grad-CAM visualizations are presented in Fig. 13. The Grad-CAM visualizations are highly correlated with where radiologists look at when making decisions. For instance, radiologists tend to concentrate on the global appearance of an image when assessing ‘noise,’ whereas local features gain more attention during evaluations of ‘diagnosis’ or ‘lesions.’

Overall, the above findings indicate that IQAGPT can successfully perform CT subjective quality-assessment tasks. It can predict texts aligned with the GT and also translate these texts into scores and reports using ChatGPT in a clinically meaningful way.

## Discussion

This study highlights the efficacy of integrating large models for IQA, with a specific focus on LDCT denoising. It suggests a significant potential to replace the traditional subjective image-quality evaluation procedure conducted by radiologists with large hybrid deep models, which are resource-efficient and time-saving. In other words, the developed IQAGPT is the first attempt in this direction, and IQAGPT not only eases the burden on radiologists by automating CT IQA but also aids radiologists in refining diagnostic performance.

 The proposed method was developed on the CT-IQA dataset of 1,000 image-text pairs annotated by a professional radiologist. For this purpose, a prompt template was leveraged to transform quality scores into text descriptions. Having fine-tuned the image-quality captioning model on the CT-IQA dataset, IQAGPT can generate quality descriptions for different CT scans. Using ChatGPT as an interactive interface facilitates user engagement, allowing for versatile outputs including quality scores and comprehensive reports.

Experimental results demonstrate the efficacy of IQAGPT in steadily generating quality descriptions and converting them into scores and reports. Quantitative evaluation using metrics for image captioning, classification, and regression tasks, underscores the superior performance of IQAGPT. In addition, ablation studies show the effectiveness of incorporating LLMs in subjective CT-IQA tasks; IQAGPT can integrate the expertise of radiologists with the advanced capabilities of LLMs. Furthermore, LLM provides an interpretation of generated results using the quality captioning model. While CLIP-IQA also employs LLMs, its limitation to training one metric at a time with simple text prompts restricts its applicability in complex medical IQA scenarios, especially when assessing fine structures and small lesions.

However, it is acknowledged that there are some limitations of the CT-IQA dataset. First, the relatively small size of the dataset might have made the training process of the LLM sub-optimal. Owing to the high cost and significant time required for professional radiologist annotations, the study aims to validate the feasibility of using LLMs for IQA, serving as a rapid communication to demonstrate the potential of the proposed approach. In the future, it is planned to collect more clinical data to conduct larger-scale experiments and further validate current findings. Although the external evaluation indicates a strong correlation between radiologists, the dataset annotated by a single radiologist still introduces a small bias into the model. In the future, it is planned to use mean calibration or small-scale fine-tuning to adapt to the preferences of different radiologists. Since IQAGPT represents the initial effort in IQA using LLMs, the reliance on the annotation standards of the prior studies may not fully encompass the complexity of image-quality nuances [28, 34], such as body parts and lesion types. In the future, text descriptions could be significantly refined, and different types of CT images could be added for IQA, thereby broadening its applicability and effectiveness in clinical scenarios.

## Conclusions

This study presents a pioneering exploration into CT subjective quality assessment, using an innovative amalgamation of VLMs and ChatGPT. We collected CT-IQA, an image-text dataset comprising pairs of CT scans with quality scores annotated by an experienced radiologist. We develop IQAGPT, fine-tuned on a VLM using the CT-IQA dataset, which can integrate with ChatGPT to generate both quality scores and detailed reports. The results of extensive experiments not only demonstrate the feasibility of IQAGPT but also highlight the effectiveness of LLMs, marking a significant potential of integrating LLMs in the field of subjective IQA.

## Data Availability

For accessing the dataset used in this paper, please contact the corresponding author Hongming Shan.

## Abbreviations

LLMs: Large language models
VLMs: Vision-language models
IQA: Image quality assessment
CT: Computed tomography
PSNR: Peak signal-to-noise ratio
SSIM: Structural similarity
RMSE: Root-mean-square error
NDCT: Normal-dose CT
LDCT: Low-dose CT
QRM: Quality reference mAs
BLEU-n: Bilingual evaluation understudy for n words
ROUGE-L: Recall oriented understudy of gisting evaluation for longest common subsequence
METEOR: Metric for evaluation of translation with explicit ordering
CIDEr: Consensus-based image description evaluation
PLCC: Pearson linear correlation coefficient
SROCC: Spearman’s rank order correlation coefficient
CLS: Classification
GT: Ground-truth
R1: Radiologist 1
R2: Radiologist 2
R3: Radiologist 3
